# Patient Journey Map: Metal Hypersensitivity

**DOI:** 10.1177/23743735231183576

**Published:** 2023-06-20

**Authors:** J. Adrienne Lagura, Dzifa Dordunoo, Anastasia Mallidou, Jett Carey, Elizabeth M. Borycki, Andre Kushniruk

**Affiliations:** 1 School of Nursing, University of Victoria, Victoria, Canada; 2 175083 School of Health Information Science, University of Victoria, Victoria, BC, Canada

**Keywords:** patient safety, patient education, implantable device, journey map, patient experience, metal hypersensitivity

## Abstract

In this study, we highlight patients’ experiences with metal hypersensitivity (MH) after receiving implantable medical devices (IMDs). We aim to identify gaps in clinical care and improve outcomes for individuals who have or may be sensitive to metals. Secondary data analysis from a previous interpretative phenomenological qualitative study was utilized. Using patient journey maps, we explored the experiences of 8 individuals from outpatient settings who received IMD and have first-hand experience with MH. We documented their journey from MH symptom recognition to diagnosis and subsequent IMD management. The results reveal that the time frames from device implantation to the treatment of MH varied from 17 to 228 months. The longest phase on the patient journey maps was the symptom recognition phase, which refers to the time between symptom emergence and MH diagnosis. Participants also required extensive healthcare utilization following their initial surgery. These findings emphasize that MH should be considered in differential diagnoses for patients with IMD. Early screening and detection of MH can enhance patient safety, alleviate distress, and reduce unnecessary healthcare utilization.

## Introduction

Metals have been widely used in society for the last 5000 years, and their utilization has been crucial for economic and societal progression.^1^ An essential characteristic of metals is their ability to combined to form “alloys”.^1^ These alloys are essential components of various life-saving medical devices that are intended to be permanently implanted in the body.^1^ Medical devices with metal components can range from simple orthopedic screws to intricate bioelectronics containing cardiac pacemakers and neurostimulators.^2^ The use of such implantable medical devices (IMDs) has been on the rise over the last few decades.^1,2^ Individuals who undergo procedures that involve IMDs can experience hypersensitive immune reactions to the metal components.^3‐5^ Metals are known to trigger Type I through IV hypersensitivity reaction, but the term metal hypersensitivity (MH) is used to describe Type IV reaction.^6^ It is believed that ions released from corrosion of the implantable device bind with protein in the blood to trigger an immune response.^6,7^ Type IV immune response differs from the other types in that it is a T-cell-mediated reaction with delayed onset and lacks localized debris from the implant degradation on histological examination.^6‐9^

The true prevalence of MH is unknown and likely underreported because the symptoms overlap with many conditions, including postsurgical infection.^10^ Postsurgical infection rate ranges from 0.5% to 3% in the presence of coordinated infection control interventions.^11^ While MH rates range from 20% to 80% without coordinated strategies to mitigate its occurrence.^5,12,13^ MH cases are also underreported due to the lack of consistent means of detecting this issue. The most common ways to diagnose MH are through in vivo testing, such as patch testing, or in vitro testing with blood assays such as memory lymphocyte immunostimulation assay (MELISA), lymphocyte transformation test, and lymphocyte proliferation testing.^14,15^ Patch testing has been widely accepted as a standard diagnostic tool among allergists for many years owing to its high sensitivity and specificity (70%-80%).^14^ Though a patch test has high sensitivity, it also has high false positive rates; therefore, a combination of tests is suggested for detecting the presence of MH.^14‐16^ Oftentimes, MH reaction is a diagnosis of exclusion, further delaying diagnosis and management of the patient's symptoms.^17,18^ Thus, MH is both a quality of care and patient safety issue that needs innovative approaches to help clinicians detect and treat symptoms in a timely manner to alleviate patient suffering.

The growing importance of patient empowerment and increased involvement in their self-care has propelled interest in mapping their journeys.^19,20^ Journey mapping can produce visual tools that incorporate both the physical and emotional aspects of a patient's journey with the goal of capturing their feelings, motivations, and attitudes across the episodes of care.^21^ Journey maps are snapshots in time that explore the mindsets, emotions, and experiences of the user.^22^ The utilization of patient journey mapping originated from software literature in the area of user mapping.^23,24^ Specifically, in healthcare, this approach highlights user experiences (eg, patients, caregivers, and healthcare providers) and perceptions around delivering and receiving care while allowing for common themes to emerge in an engaging graphic illustration. In the past, patient journey mapping has also been successfully utilized to inform policy direction and help advance patient-centered cancer care as well as improve outpatient medical appointments.^24‐26^ As patient journeys are mapped, patterns, barriers, and gaps in healthcare delivery are identified from a patient-centered perspective.^20^ Likewise, challenges that exist due to the gaps in knowledge about screening, diagnoses, and management can be better visualized through emerging themes on the journey map.^27^ Overall, the utilization of patient journey maps is a great approach to supporting shared decision-making, decreasing fragmented patient care, and encouraging patient empowerment that fosters positive patient outcomes.^28^ In this paper, we explore patients’ experiences of MH using patient journey maps to inform strategies to help overcome the fragmentation in the health system that allows delays in the diagnosis and management of MH.

## Methods

### Methodological Overview

This study is a secondary data analysis of transcribed interviews from a prior interpretative phenomenological qualitative study aimed at deciphering what information would help patients make an informed decision about metal implants.^29^ The original study included individuals with a history of MH who had undergone procedures with metal implants.

### Recruitment

The methods have been previously reported (29) but briefly, after ethics approval was obtained (University of Victoria, #19-0473), the original study used a snowball sampling approach to enroll 16 participants with lived experiences of MH using various patient recruitment methods, including social media, patient partners, and the provincial patient recruitment platform. Participation was voluntary, and the participants received a gift card with the value of $50 in their local currency. This secondary analysis included 8 participants with orthopedic IMD whose stories had sufficient detail to allow for the construction of a patient journey map.

### Data Collection

In the semistructured, in-depth interviews, participants were asked open-ended questions to encourage them to reflect and talk freely about their experiences with MH. Additional prompts about the consent process, along with questions and probes about the diagnosis and management of MH were asked (Supplemental Appendix). These in-depth conversations were the main data source for this analysis. The interviews were conducted in English, took place on a virtual commercial platform, and were audio-recorded. The audio recordings were transcribed by commercial software, verified against the audio by a research assistant, deidentified, then saved on a secure university password-protected server and computer. The audio recordings were deleted once the transcriptions were verified by the participants. Field notes and transcript interviews were likewise anonymized.

### Mapping Process

The approach of using patient journey mapping was inspired by the previous work of Borycki et al.^22^ We started the mapping process by precoding the transcribed interviews and highlighting the passages or concepts that were considered significant to diagnosis and management. We developed a more formal coding scheme with the use of a Microsoft spreadsheet as a data extraction form to gather all pertinent participant demographic information. We then utilized the UXpressia® journey mapping tool to better organize the collected data, as well as to highlight patterns of knowledge gaps and fragmentation in care which led to the persistence and worsening of MH.

**Figure 1.**
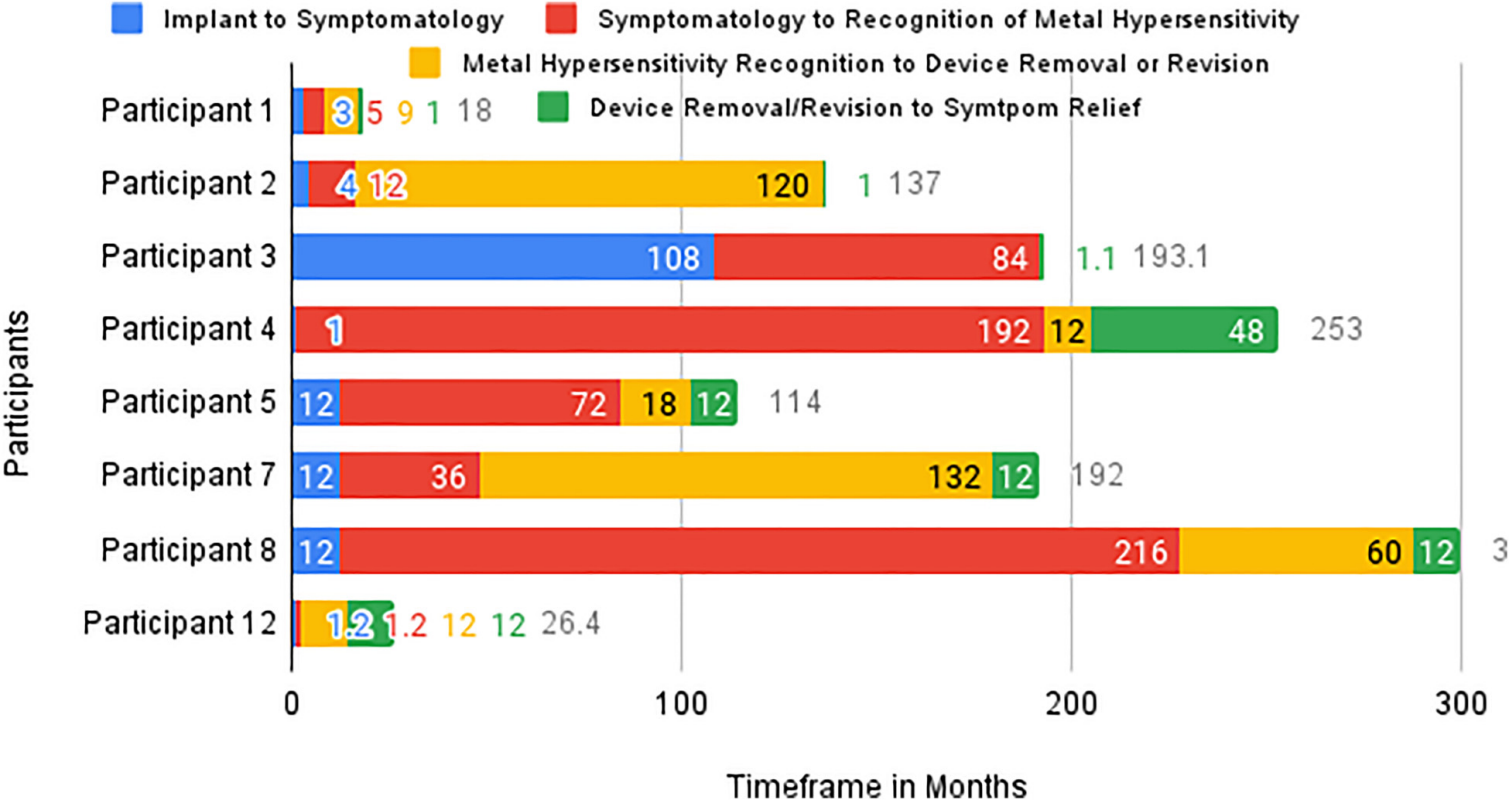
Participants’ metal hypersensitivity (MH) timeline comparison.

The authors realized that it was challenging to collectively compare the timeline information of an individual participant's journey using UXpressia®. We, therefore, utilized an Excel spreadsheet to put the data together. Following this, we converted the Excel sheet into a stacked bar chart to visualize the time frames (Figure 1). The *X*-axis highlights the participants’ journey in months, whereas the *Y*-axis represents the 8 participants that were interviewed. Specifically, the *X*-axis reflects the color-coded guiding questions asked of the participants during the interview process. Using the diagnosis reasoning as a framework, we divided the patient journey into different time points (eg, implant to symptom onset, symptomatology to recognition of MH, MH recognition to device removal, and device removal to symptom relief) to highlight time frames within the patient journey that have the longest delay. Symptomatology is defined as “the combined signs, markers, or indications of a disease or disorder”.^30^

**Figure 2.**
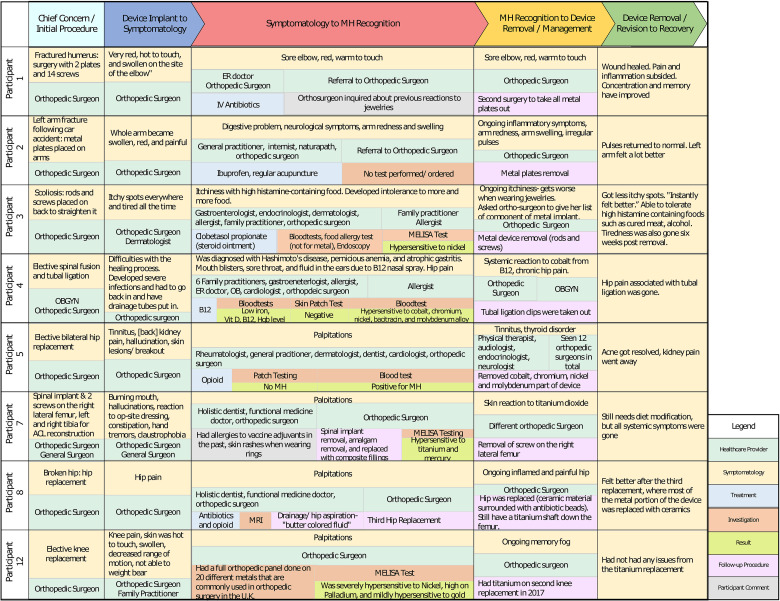
Comparison of participants’ metal hypersensitivity (MH) journey.

**Figure 3.**
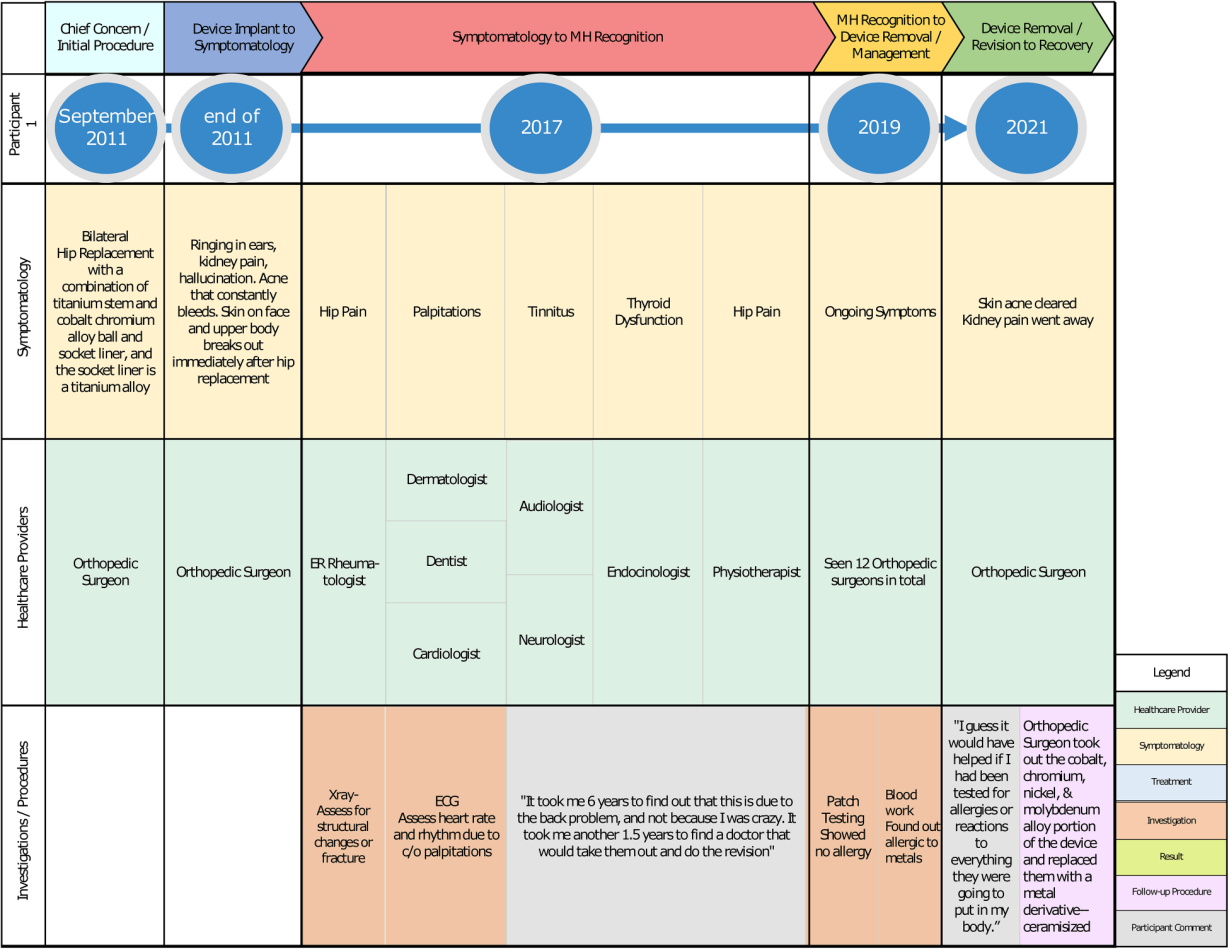
Individual metal hypersensitivity (MH) journey.

The authors used Microsoft Visio to map out each participant's journey. We summarized each participant's symptomatology with corresponding health provider visits, investigations ordered, treatment rendered, and procedures performed in Figure 2. The goal for this is to emphasize patterns of healthcare utilization and treatment delay. We also used Microsoft Visio to provide an example of an individual participant's journey from the time of device implant leading to the device removal or revision and recovery (Figure 3). This method highlights the participant's unique journey, which focuses on the timeline of events postdevice implantation experience, symptomatology, healthcare providers, and health services accessed, as well as the patient's concerns along the process.

## Results

### Participants

The sample consisted of 8participants—7 females and 1 male. Their age ranges between 34 and 65 years, with a median age of 49 years (Table 1). Three participants live in Canada, 3 in the United States, 1 in New Zealand, and 1 in the United Kingdom. The types of metals that participants were sensitive to included titanium, nickel, gold, silver, molybdenum, manganese, and cobalt. Individual participants saw between 2 and 12 different specialties during the diagnosis process. The indications for surgery included fractures following motor vehicle accidents, falls, degenerative issues, and correction of medical conditions such as spine deformities (eg, scoliosis). The surgical procedures performed were orthopedic surgery, such as internal fixation, hip replacement, spinal fusion, and anterior cruciate ligament (ACL) reconstruction. One participant also had tubal ligation performed. 

**Table 1.** Participants’ Demographics (*N* = 8).

Interview No.	Age	Sex	Race	Country	Procedure performed	Type of metal hypersensitivity	Healthcare provider seen
1	58	Female	White	CAN	Internal fixation with plates and screws	Titanium	Two Orthopods Physiotherapist Physiotherapist ER Doctor
2	65	Female	White	CAN	Internal fixation with plates	Unknown	Orthopedic Surgeon Family Physician ER Doctor Naturopath Rheumatologist Internist
3	34	Female	White	USA	Spinal fusion	Nickel	Dermatologist Orthopedic Surgeon Gastroenterologist Endocrinologist Allergist Family Physician
4	48	Female	White	USA	Spinal fusion, tubal ligation	Titanium	Orthopedic Surgeon OBGYN ER Doctor Gastroenterologist Allergist Family Physician Cardiologist
5	40's	Female	White	USA	Bilateral hip replacement, bilateral hip revision	Cobalt, molybdenum	Rheumatologist Dermatologist Multiple Orthopedic Surgeons ×12 Dentist Cardiologist Physical Therapist Audiologist Endocrinologist Neurologist ER Doctor Family Physician
7	48	Male	N/A	NZL	Spinal implant, anterior cruciate ligament (ACL) reconstruction	Titanium, mercury	Orthopedic Surgeon General Surgeon Holistic Dentist Functional Medicine Doctor
8	59	Female	White	CAN	Hip replacement ×3	Titanium	Orthopedic Surgeon Neurologist ER Doctor
12	55	Female	N/A	UK	Left knee replacement	Nickel, palladium, sterling	Orthopedic Surgeon ×2 Family Doctor

### Timeline

Using the information, we captured the time frames from when participants first noticed their MH-related symptomatology to diagnosis, then the period between diagnosis and device removal, and revision or replacement with an alternative material (Figure 1). This allowed us to highlight the gaps in healthcare diagnosis and management by evaluating the length of time before their health providers recognized that the symptoms were related to the IMDs.

The time frames from device implantation to the treatment of MH range from 17 to 228 months (19 years). Treatment of MH for most of the participants was either implant removal (eg, implant completely removed), replacement with a device made of hypoallergenic material, such as ceramics, or revision with partial removal of the offending implant. The person with the shortest time frame had surgery for a fractured humerus. Her symptos were first noticed 3 months after the surgery (Figure 1). She saw 2 orthopods prior to the IMD removal. On the other hand, the person with the longest time frame had an IMD with a metal composition placed in 1995 for a hip replacement (Figure 1). She had revision surgery when MH was diagnosed, but some parts of the device remain in situ. The participant stated that

*I still have a titanium shaft down the femur. That's the original from nineteen ninety-five* [which could not be removed because] *you'd be destroying the femur if you tried to* [completely] *remove that titanium shaft right down the middle there*.

Comparing the journey of the 8 participants, the longest period on the patient journey map is the symptomatology to MH recognition phase, defined as the time between the emergence of symptoms to the time of MH diagnosis. The duration of this phase ranges from 1.5 to 216 months. On the other hand, the shortest time frames on the patient journey map are the period between device implant to symptom onset and treatment of MH (removal, revision, or replacement), and symptom relief. This ranges from 1 to 108 months.

### Manifestations

The signs and symptoms following the device implant include: swelling, redness, pain, abscess to the previous surgical site, tinnitus, kidney pain, skin lesions, breakout, decreased range of motion, delay in wound healing, and hallucinations (Figure 2). In addition to localized signs and symptoms such as chronic pain, redness, and swelling in the surgical area. Participants also developed systemic signs and symptoms such as digestive problems, intolerance with high histamine-containing foods, burning mouth, palpitations, and neurological concerns such as brain fog, hallucinations, claustrophobia, and hand tremors. The health system utilization may be influenced by the nonspecific symptomatology of MH.

### Health System Utilization

Based on the patient journey map, participants had high healthcare utilization following their index surgery, particularly during the symptomatology to MH recognition phase (Figure 1). Throughout this period, each participant sought various healthcare providers including orthopedic surgeons, family practitioners, allergists, endocrinologists, holistic dentists, rheumatologists, dermatologists, cardiologists, and internists. Participant 5, in particular, saw 12 different orthopedic surgeons from the time of the index surgery to the time she had her metal hip implant replaced with ceramic (Figure 2). Furthermore, all participants sought help from a minimum of 3 health professionals of various specialties for the same presenting concern. During their healthcare visits, various diagnostics were ordered, including blood work to assess for infection, magnetic resonance imaging, computed tomography scan, x-rays, and endoscopy; however, only 2 providers ordered MH tests (MELISA test and skin patch test) at the initial visit following the presence of their symptomatology. Eventually, 5 participants were also tested for MH via a skin patch test or blood work; however, this was performed after several other investigations, treatments, and several visits to various specialists (Figure 2). Moreover, procedures performed include incision & drainage of the inflamed area, spinal implant removal, redo hip and knee replacement, removal of metal plates or IMDs, removal of tubal ligation clips, and removal of offending metal parts of the device. Complementary treatments include acupuncture, physiotherapy, and diet modification. They also received pharmacological treatments including antibiotics, opioids, and steroids.

To further show how the delay of MH recognition affects each individual, we looked closer into Participant 5's experience (Figure 3). The participant had a bilateral hip replacement in 2011. Following her surgery, she developed symptoms such as rashes, ringing in her ears, kidney pain, and hallucinations. She saw various health professionals such as family practitioners, rheumatologists, dermatologists, dentists, cardiologists, physical therapists, audiologists, endocrinologists, neurologists, and multiple orthopedic surgeons. She mentioned that

…it took me six years to find out that this is due to the back problem, and not because I was crazy. It took me another 1.5 years to find a doctor that would take them out and do the revision.

She found out through her own research that she might be sensitive to metals; therefore, she facilitated getting a blood test which confirmed that she was hypersensitive to cobalt and molybdenum. Ten years following the initial surgery, the patient eventually had that portion of IMD taken out in 2021. Her symptoms resolved following the device removal. Reflecting on her experience and suggestions for improvement, she said “I guess it would have helped if I had been tested for allergies or reactions to everything they were going to put in my body.”

## Discussion

The journey map shows a wide variation in duration from the onset of symptoms to treatment and management for MH. This appears to be due to a lack of awareness among healthcare providers about the signs and symptoms of metal toxicity which is fueled by the nonspecific symptomatology of MH.^3^ Participant 5 in this study reported symptoms that are consistent with cobalt toxicity, which can include hearing sensorineural hearing loss, vision loss, cognitive decline, cardiomyopathy, hypothyroidism, weakness, fatigue, and muscle spasticity.^31^ These symptoms can also be due to other disease processes making the diagnosis process of MH difficult. It has been suggested that MH can occur between 2 months and 2 years after a joint replacement.^6^ Our findings are consistent with the reported literature.^6,31^ The longest delays appear to be arriving at the diagnosis of MH and getting treatment for MH. This variation in timeline suggests a need to implement postimplant surveillance to capture issues arising. Currently, there is no consensus in the literature about when on the patient journey issues of MH may occur. In addition, there are no standardized criteria for when to consider MH reaction. Rosner and Fonacier^32^ discussed the diagnostic criteria for MH reactions postimplantation, which include major and minor symptoms, however, the authors did not specify how many of the the minor and major criteria are required for the diagnosis of MH. Therefore, more research is needed to pinpoint the trajectory of corrosion, symptoms onset, and associated time frame to guide the surveillance period.

The study participants used various methods to test for MH, such as blood tests, MELISA, and skin patch testing. It is also important to note that 2 participants tested negative for MH via patch testing; however, their blood tests showed MH. This suggests the need for further research on the specificity and sensitivity of MH tests.^11^ Patients also underwent procedures, tests, and treatments that were not indicated for MH (eg, antibiotics therapy) as well as some patients reported having seen more than 10 specialists. This suggests the diagnosis of exclusion approach to MH leads to potential unnecessary healthcare utilization. More studies are needed to help determine the cost-effectiveness of postoperative surveillance compared to the status quo and high healthcare utilization. The patient journey map also demonstrates a lack of continuity of care among healthcare professionals. Specifically, although patients saw many healthcare providers, it is unclear what information was communicated among each other.

Our findings suggest symptom recognition of MH is hindered by a lack of knowledge about MH. Our previous study suggests patients received inadequate information about MH during the consenting process thus when they developed symptoms, they were unsure about the possible causes.^29^ Healthcare providers such as nurses, surgeons, general practitioners, and those in outpatient settings need to be aware that this condition is associated with high morbidity and healthcare utilization. Inquiring about MH during initial history taking and/or subsequent healthcare visits is a good initial step. Integrating information about MH into hospital or clinic learning hubs and resource websites is another way to enhance the knowledge transfer to healthcare providers. In addition, providers need to consider this among the differential diagnosis of patients with metal implantable devices presenting postoperatively for evaluation. The journey map indicated participants who were referred to allergists had metal allergy tests. This suggests engaging an immunologist early in the patient journey might be helpful in establishing the diagnosis of MH earlier. Given that participants encountered difficulties identifying the reason for their presenting concerns, which led them to seek multiple healthcare services and undergo various diagnostic testing, there is a need to use health information technologies such as digital apps that patients can use to record and report emerging symptoms to their primary provider with clinical expertise in immunological reactions to implantable devices. Integrating such apps into electronic health records may help providers arrive at a diagnosis earlier.^33^

### Strengths and Limitations

A strength of our study is that we collected data from patients across different countries who had undergone surgeries involving metal implants (eg, hip replacement, spinal fusion, and ACL reconstruction). This allowed us to capture and compare patients’ journeys with MH across different settings. In addition, to the best of our knowledge, this is the first study to use a patient journey map to visualize people's experience of MH. This study also provided an opportunity for the participants to have a voice and discuss their experiences as our previous literature review failed to identify any qualitative studies that detailed the patient experience from their perspective.^34^

The main limitation of this study is the lack of comprehensive health utilization data to determine the economic burden of MH. The use of retrospective data is prone to recall bias while the use of snowballing sampling could result in selection bias. Another limitation is the small sample size (*n* = 8). Sixteen people took part in the original study; however, some interviews lack specificity about the timeline and were excluded. Lastly, although some participants had copies of their medical records, part of the challenge was that not all participants had access to their own medical information due to paper charting and limited use of health information technology.

## Conclusion

Metals have been widely utilized in healthcare due to the variety of uses it serves, such as being an essential component of medical devices. However, along with the benefits, it is important to consider the adverse event such as MH. The use of the patient journey map of MH reveals extended delays in symptom recognition, diagnosis as well as management phases. Therefore, having an early MH screening and detection tool can help promote patient safety, lessen the burden of distress the patient might experience, and save on potentially unnecessary health system utilization. There is no consensus in the literature about the diagnostics approach for MH; thus, more attention to the clinical tests for MH is needed. Furthermore, exploring how health information technologies can be used to record and report emerging symptoms to their primary provider with clinical expertise in immunological reactions to implantable devices may help providers arrive at a diagnosis sooner. With the current shortage of healthcare providers and pressure on health services, which are made worse by the Covid-19 pandemic, preventing unnecessary complications that people with MH could experience after their device implant can help alleviate the burden in our healthcare system. To this end, the journey map we created can be used to start a conversation that acknowledges the existence of MH is a step closer to promoting safety while more research about MH is generated, with the aim to positively impact patient outcomes, as well as reduce morbidity and associated healthcare costs locally and globally.

## Supplemental Material

sj-docx-1-jpx-10.1177_23743735231183576 - Supplemental material for Patient Journey Map: Metal Hypersensitivity Click here for additional data file. Supplemental material, sj-docx-1-jpx-10.1177_23743735231183576 for Patient Journey Map: Metal Hypersensitivity by J. Adrienne Lagura, Dzifa Dordunoo, Anastasia Mallidou, Jett Carey, Elizabeth M. Borycki and Andre Kushniruk in Journal of Patient Experience

## References

[1] Cutler CP . Use of metals in our society. In: Chen JK Thyssen JP , eds. Metal allergy. Springer International Publishing; 2018:3‐16.

[2] Badhe RV Akinfosile O Bijukumar D Barba M Mathew MT . Systemic toxicity eliciting metal ion levels from metallic implants and orthopedic devices—a mini-review. Toxicol Lett. 2021;350:213‐24. doi:10.1016/j.toxlet.2021.07.004 34252509

[3] Dordunoo D Hass M Smith C , et al. Metal hypersensitivity screening among frontline healthcare workers—a descriptive study. J Clin Nurs. 2021;30(3–4):541‐9. doi:10.1111/jocn.15571 33237599

[4] Lhotka CG Szekeres T Fritzer-Szekeres M , et al. Are allergic reactions to skin clips associated with delayed wound healing? Am J Surg. 1998;176(4):320‐3. doi:10.1016/s0002-9610(98)00197-4 9817247

[5] Hallab N Merritt K Jacobs JJ . Metal sensitivity in patients with orthopaedic implants. J Bone Joint Surg. 2001;83-A(3):428‐36. doi:10.2106/00004623-200103000-00017 11263649

[6] van der Merwe JM. Metal hypersensitivity in joint arthroplasty. J Am Acad Orthop Surg. 2021;5(3):e20.00200. doi:10.5435/JAAOSGlobal-D-20-00200 PMC7963506 33720103

[7] Hallab N Merritt K. Current concepts review: metal sensitivity in patients with orthopaedic implants. J Bone Jt Surg Am. 2001;83-A(3):428‐36. 10.2106/00004623-200103000-00017 11263649

[8] Eliaz N . Corrosion of metallic biomaterials: a review. Materials (Basel). 2019;12(3):407. doi:10.3390/ma12030407 30696087 PMC6384782

[9] Büdinger L Hertl M. Immunologic mechanisms in hypersensitivity reactions to metal ions: an overview. Allergy*.* 2000; 55(2), 108‐15. doi:10.1034/j.1398-9995.2000.00107.x 10726725

[10] Anand A McGlynn F Jiranek W . Metal hypersensitivity: can it mimic infection? J Arthroplasty. 2009;24(5):826.e25‐e28. 10.1016/j.arth.2008.05.002 18639432

[11] Seidelman JL Mantyh CR Anderson DJ . Surgical site infection prevention: a review. JAMA. 2023;329(3):244‐52. 36648463 10.1001/jama.2022.24075

[12] Thyssen JP Linneberg A Johansen JD . The epidemiology of contact allergy in general population-prevalence and main findings. Contact Dermatitis. 2007;57(5):287‐99. doi:10.1111/j.1600-0536.2007.01220.x 17937743

[13] Zondervan RL Vaux JJ Blackmer MJ Brazier BG Taunt J. Improved outcomes in patients with positive metal sensitivity following revision total knee arthroplasty. J Orthop Surg Res. 2019;14(1):182. doi:10.1186/s13018-019-1228-4 31208448 PMC6580588

[14] White JM . Patch testing: what allergists should know. Clin Exp Allergy. 2012;42(2):180‐5. doi: 10.1111/j.1365-2222.2011.03862.x 22092850 10.1111/j.1365-2222.2011.03862.x

[15] Brace DN Hegde V Johnson R Kleeman-Forsthuber L Jennings J Douglas D. Poor correlation among metal hypersensitivity testing modalities and inferior patient-reported outcomes after primary and revision total knee arthroplasties. Arthroplasty Today*.* 2022;18:138‐42. doi:10.1016/j.artd.2022.09.016 36345325 PMC9636001

[16] Caicedo M . Metal hypersensitivity to implant materials. TMJ Association; 2021. Accessed July 2022. https://tmj.org/living-with-tmj/tmj-implants/metal-hypersensitivity/.

[17] King SW Royeca JM Cunningham CM Madegowda R Sha S Pandit H. Metal hypersensitivity in total knee arthroplasty. J Arthrosc Joint Surg*.* 2020;7(4):184‐8.

[18] Innocenti M Vieri B Melani T Paoli T Carulli C. Metal hypersensitivity after knee arthroplasty: fact or fiction? Acta Biomed. 2017; 88(2S):78‐83. doi:10.23750/abm.v88i2-S.6517 28657568 PMC6178998

[19] Eysenbach G Diepgen TL . The role of e-health and consumer health informatics for evidence-based patient choice in the 21st century. Clin Dermatol. 2021;19(1):11‐7. doi:10.1016/S0738-081X(00)00202-9 11369478

[20] Davies EL Bulto LN Walsh A , et al. Reporting and conducting patient journey mapping research in healthcare: a scoping review. J Adv Nurs. 2023;79(1):83‐100. doi:10.1111/jan.15479 36330555 PMC10099758

[21] McCarthy S O’Raghallaigh P Woodworth S Lim YY Kenny LC Adam F . Embedding the pillars of quality in health information technology solutions using “integrated patient journey mapping” (IPJM): case study. JMIR Hum Factors. 2020;7(3):e17416. doi:10.2196/17416 32940610 PMC7530692

[22] Borycki EM Kushniruk AW Wagner E Kletke R. Patient journey mapping: integrating digital technologies into the journey. Knowl Manage E-Learn*.* 2020; 12(4): 521‐35. doi:10.34105/j.kmel.2020.12.029

[23] Kalbach J. Mapping experiences: a guide to creating value through journeys, blueprints, and diagrams. O’Reilly*;* 2016.

[24] BC Patient Safety and Quality Council. Journey mapping in cancer care. BC Patient Safety and Quality Council; 2019. Accessed April 2022. https://bcpsqc.ca/wp-content/uploads/2019/01/Journey-Mapping-Cancer-interactive.pdf

[25] Jones PH Shakdher S Singh P . Synthesis maps: visual knowledge translation for the CanIMPACT clinical system and patient cancer journeys. Curr Oncol 2017;24(2):129‐34. doi:10.3747/co.24.3452 28490928 PMC5407865

[26] Clinica Las Condes. Patient journey maps: Clinica las condes (CLC). Accessed June 12, 2022. https://www.hopkinsmedicine.org/international/partners-forum/past-presentations/2016/04_boekemeyer_slater_mapping_the_patient_experience_at_clc.pdf

[27] Kushniruk AW Borycki EM Parush A. A case study of patient journey mapping to identify gaps in healthcare: learning from experience with cancer diagnosis and treatment. Knowl Manage E-Learn. 2020; 12(4): 405‐18. doi:10.34105/j.kmel.2020.12.022

[28] Joseph AL Kushniruk AW Borycki EM. Patient journey mapping: current practices, challenges and future opportunities in healthcare. Knowl Manage E-Learn*.* 2020; 12(4):387‐404. doi:10.34105/j.kmel.2020.12.021

[29] Dordunoo D Doane G Carey J , Lagura JA, Mallidou A, van der Merwe J, Schroeder S. The lived experience of metal hypersensitivity: a qualitative study. [submitted].

[30] American Psychological Association. APA dictionary of psychology. American Psychological Association. Accessed February 15, 2023. https://dictionary.apa.org/

[31] Venkatraman V Megan KW Chidyaonga S Beth P Shivanand PL . Cobalt-induced toxicity and spasticity secondary to hip arthroplasty: case report and review of the literature. Curēus. 2020;12(12):e12368. doi:10.7759/cureus.12368 33527049 PMC7842236

[32] Rosner GA Fonacier LS . Hypersensitivity to biomedical implants: prevention and diagnosis. Allergy Asthma Proc. 2017;8(3):177‐83. doi:10.2500/aap.2017.38.4052 28441987

[33] Opoku-Agyemang E Dordunoo D Ahmelich A , et al. Patient safety and health information technology conceptual framework. Knowl Manage E-Learn. 2021;13(4):395‐407.

[34] Dordunoo D Anaman-Torgbor J Smith C , et al. Hypersensitivity in patients receiving metal implants: a scoping review protocol. JBI Evid Synth. 2021;19(6):1404‐1411. 33278266 10.11124/JBIES-20-00171

