# Supplementary material for: Patient Journey Map: Metal Hypersensitivity
Source: J Patient Exp. 2023 Jun 20;10:23743735231183576. doi: 10.1177/23743735231183576 (PMC10286172; doi:10.1177/23743735231183576)
Supplement: sj-docx-1-jpx-10.1177_23743735231183576 - Supplemental material for Patient Journey Map: Metal Hypersensitivity [file sj-docx-1-jpx-10.1177_23743735231183576.docx]

# Interview Guide

Study: The lived experiences of metal hypersensitivity

Research question:

**Among patients with metal hypersensitivity**, **what information would help them make an informed decision about metal implants**

Tell me about your experience of how you came to know that you have allergy to metal or metal hypersensitivity.

Tell me about your experience with the health system

How was your situation addressed? What was done to alleviate your condition?

# Additional prompts:

What question would have been helpful for the healthcare worker(s) to ask you to trigger you to disclose your history of metals?

What about the experience stand out in your mind?

What was the single most important thing you think can be done to make this situation better in the future?

What sort of things would you like the healthcare system to know about how they interact with you or other patients with this issue?

If you could live the experiences again, would you change anything? If so, what? If no, why? Is there anything else you want to tell me about your experience?
